# Treatment of *E. coli* Infections with T4-Related Bacteriophages Belonging to Class *Caudoviricetes*: Selecting Phage on the Basis of Their Generalized Transduction Capability

**DOI:** 10.3390/v17050701

**Published:** 2025-05-14

**Authors:** Alexandra N. Nikulina, Nikita A. Nikulin, Natalia E. Suzina, Andrei A. Zimin

**Affiliations:** 1 Laboratory of Molecular Microbiology, G.K. Skryabin Institute of Biochemistry and Physiology of Microorganisms, Federal Research Center “Pushchino Scientific Center for Biological Research of the Russian Academy of Sciences”, 142290 Pushchino, Russia; n.nikulin@ibpm.ru (N.A.N.); dr.zimin8@yandex.ru (A.A.Z.); 2 Laboratory of Cytology of Microorganisms, G.K. Skryabin Institute of Biochemistry and Physiology of Microorganisms, Federal Research Center “Pushchino Scientific Center for Biological Research of the Russian Academy of Sciences”, 142290 Pushchino, Russia; suzina_nataliya@rambler.ru

**Keywords:** phage therapy, generalized transduction, horizontal gene transfer, T4-related phages

## Abstract

The problem of the multidrug resistance of pathogenic bacteria is a serious concern, one which only becomes more pressing with every year that passes, motivating scientists to look for new therapeutic agents. In this situation, phage therapy, i.e., the use of phages to combat bacterial infections, is back in the spotlight of research interest. Bacterial viruses are highly strain-specific towards their hosts, which makes them particularly valuable for targeting pathogenic variants amidst non-pathogenic microflora, represented by such commensals of animals and humans as *E. coli*, *S. aureus*, etc. However, selecting phages for the treatment of bacterial infections is a complex task. The prospective candidates should meet a number of criteria; in particular, the selected phage must not contain potentially dangerous genes (e.g., antibiotic resistance genes, genes of toxins and virulence factors etc.)—or be capable of transferring them from their hosts. This work introduces a new approach to selecting T4-related coliphages; it allows one to identify strains which may be safer in terms of involvement in the horizontal gene transfer. The approach is based on the search for genes that reduce the frequency of genetic transduction.

## 1. Introduction

*Escherichia coli* is a commensal of humans and animals that inhabits the gastrointestinal tract. Some *E. coli* strains, however, are pathogenic and can cause severe disorders, such as hemorrhagic colitis, acute diarrhea, urinary tract diseases, neonatal meningitis, sepsis and avian aerosacculitis. The measures to prevent *E. coli* infections include various sanitary procedures, control for the quality of food and drinking water, vaccination, and the use of probiotics—with the treatment of the infections mostly based on the administration of antibiotics [1]. However, due to horizontal gene transfer and the uncontrolled use of antibacterial drugs in healthcare and animal/poultry farming, many bacterial strains quickly acquire resistance to multiple classes of antibiotics.

For example, there have been reports of *E. coli* strains becoming resistant to β-lactams, quinolones, aminoglycosides, sulfonamides and fosfomycin [2,3]. It has also been shown that, in developing countries, the strains of *E. coli* dominating the gastrointestinal tract of humans and animals contain a gene of β-lactamase AmpC [4]. Research data from 2019 to 2024 indicate that between 42% and 98% of uropathogenic *E. coli* isolates obtained from patients in various countries (both with middle and low income levels) may exhibit multiple antibiotic resistance [2,3].

In this regard, the use of bacterial viruses, bacteriophages, for the prevention and treatment of bacterial infections has once again become popular. As demonstrated in the studies conducted on mice and birds, phage cocktails rival antibiotics in terms of efficiency. Let us look, for example, at the data obtained in the experiments with mice infected with STEC O157: H7 EDL933. The infected animals were treated with either enrofloxacin or a phage cocktail and, while mortality-wise enrofloxacin was slightly more effective (0 versus 10%, respectively), phage-treated mice recovered and gained weight faster. In addition, the amount of pathogen in the feces of phage-treated mice dropped significantly as early as the 2nd day of treatment (in the case of enrofloxacin, it took 4 days to reach that point [5]).

Selecting phages for the treatment of bacterial infections is a complex task and requires an integrated approach. It is particularly important that the selected phage does not contain potentially dangerous genes (e.g., antibiotic resistance genes, genes of toxins and virulence factors), which can be transferred from its host [6,7]. With this in mind, researchers usually choose lytic phages over temperate ones, as the latter can integrate into the bacterial genome during infection and induce a horizontal gene transfer by the mechanism of specific transduction. It has also been discovered that phages of *Staphylococcus aureus* can initiate another type of transduction, i.e., lateral transduction. This type of transduction is found to occur with a rather high frequency [8]. In contrast to temperate ones, phages with a lytic development cycle can only participate in horizontal gene transfer by the mechanism of generalized transduction, and the frequency of this type of transduction is quite low. It should be noted, however, that generalized transduction can lead to significant adaptive changes in host bacteria (e.g., acquisition of antibiotic resistance genes [9]). Of course, the safety rules for the preparation of phage cocktails are aimed at excluding the possibility of foreign DNA contamination and minimize the risks of transduction: they prescribe the use of laboratory strains of bacteria, free (or, at least, almost free) of plasmids and prophages, virulence factors, etc. However, the frequency of horizontal gene transfer by the selected phages in the patient’s body is typically unknown. Meanwhile, if the transfer affects genetic traits that dramatically increase the fitness of bacteria, the traits can rapidly spread in the population [9]. The codex of safety rules for the selection of therapeutic phages (adopted in 2015) leaves the requirement of being non-transducing as optional, as it is currently impossible to exclude the possibility of a therapeutic phage to perform a generalized transduction of genetic information from a pathogen to a commensal strain or vice versa [6]. Hence, the search for phages whose ability to transfer genetic information would be as low as possible is a priority goal for both ensuring the safety of patients and preventing the appearance of new pathogenic strains.

As of today, the number of identified bacteriophages capable of infecting *E. coli* is quite large. Among them is the group of so-called T4-related phages. T4-related phages are phages that are morphologically and phylogenetically similar to phage T4; in particular, having homologs of its major capsid protein, a product of the *23* gene of the phage. T4-related phages have a wide range of hosts, and many are capable of infecting *Enterobacteria*, including *E. coli*. The latter are referred to as coliphages. According to the modern virus taxonomy, most T4-related phages belong to the family *Straboviridae*, yet the group also have representatives from other *Caudoviricetes* taxa [10]. T4-related bacteriophages have a lytic development cycle, do not integrate into the genome of their hosts during infection, and lack bacterial virulence genes. Their biology is well studied, and their genomes are widely represented in international databases [11,12]. The cocktails of T4-related phages have been tested on various animals in a large number of in vivo studies. For example, a cocktail of T4-related coliphages belonging to the genera *Tequatrovirus* and *Mosigvirus* was found to be effective enough to stop the development of *E. coli* infection in chicken embryos and to reduce mortality to 10% [13]. Promising results were also obtained in the experiments by Huff et al., who tested a preparation of T4-related coliphage on chickens infected with *E. coli*. Depending on the route of administration, the preparation was able to reduce the mortality of the birds to 7–20% [14,15]. The cocktails of T4-related phages were successfully used on other animals as well: mice, sheep and calves [16]. Coliphages, including T4-related bacteriophages, have recently been frequently used to treat urinary tract infections caused by *E. coli* in both animals and humans [17,18]. The tests were partially successful, with the efficacy of the therapy comparable with that of antibiotics [19]. For example, a phage cocktail was successfully used to treat a 56-year-old patient with an *E. coli* infection producing beta-lactamase [20].

Coliphages have also been used to treat children hospitalized with acute diarrhea in Bangladesh. The study showed that the cocktail preparation was safe: administered orally, it did not cause any side effects, nor worsened the condition of the patients. At the same time, it could not be called efficacious. The potential reasons for the failure of this clinical trial were (1) that no antacid was provided, so the orally-administered phages may have been destroyed in the stomach; (2) that phage cocktails were not adapted to the locally-circulating *E. coli* strains; and (3) that *E. coli* titers in the intestinal tract were too low to sustain a sufficient phage replication [12].

Overall, considering their safety and efficiency, as well as a large body of knowledge about them, T4-related bacteriophages show, in our opinion, great potential in the treatment and prevention of *E. coli* infections. There are some concerns, however, based on the data of in vitro studies that indicate that, using the mechanism of generalized transduction, T4-related phages can transfer plasmids between *E. coli* strains at a fairly high frequency. In particular, a number of studies have been conducted on in vitro transduction and cotransduction of various plasmids by T4-related bacteriophages RB42, RB43, RB49 [21,22]. It has been shown that RB42, RB43, and RB49 were able to transfer the plasmid pBR322 with a frequency of ~10^−6^, and the plasmid pBR325 with a frequency of ~10^−7^. The experiments were conducted under similar conditions using the same strain, *E. coli Be*. It is worth noting that RB42, RB43, and RB49 yielded transductant colonies resistant to selective antibiotics, unlike the control phage RB8. RB8 belongs to the genus *Tequatrovirus*, a genus of phages that includes phage T4, for which the transduction of plasmids or chromosomal elements with high frequency (>10^−8^) has not been demonstrated [23]. *Tequatrovirus* phages are very similar to each other. They have no differences in the known genes, which could affect the frequency of transduction. Experiments have also been conducted to study the difference in transduction frequency depending on the conditions of its implementation (different pH, temperature, long-wave UV). Thus, it has been shown that phage RB49 can transfer the plasmid pTurboGFP-B with a frequency of ~10^−5^–10^−6^ under normal conditions, the frequency can increase depending on the effect of temperature or long-wave UV [22,24].

In general, the packing of DNA into the capsid of a phage is mediated by a special phage protein, terminase, with the functions of DNA recognition and packing initiation carried out by the small subunit of the enzyme. In phage P22, for example, the small subunit of terminase recognizes *pac* sites on the phage genomic DNA. If the host bacterium has a sequence in its genome which is homologous to *pac* (a pseudo-*pac* site), then terminase can bind to this sequence, and the bacterial DNA will be packed into the phage capsid, creating conditions for the subsequent transfer of genetic information by the mechanism of generalized transduction [8].

In case of T4-related phages, such as RB43, RB49 and RB42, the precise molecular mechanism of DNA packing into the phage capsid is still unclear. Most likely, it is similar to the packaging mechanism of the T4 phage and follows the headful packaging mechanism type. As in the case of the well-studied T4 phage, the small (gp16 or TerS) and large subunits of terminase (gp17 or TerL) are responsible for recognition and packaging of the genome. Much research has been undertaken on the work of T4 phage DNA packaging but much still remains unknown. Thus, in various sources there are contradictory data on the *pac*-site for the T4 phage. Early experimental studies have shown that the T4 DNA packaging system in vitro can efficiently package foreign DNA [25]. Later studies have shown that TerS preferentially binds to a region of its gene to initiate packaging, i.e., the *pac*-site of the T4 phage may be a GC-rich region at the 3′ end of gene *16* [26,27].

However, there is an opinion that the genome of T4 and other T4-related phages does not contain unique *pac* sites, or that the interaction with them is not strictly specific. This is mentioned in many reviews and in recent papers concerning studies of T4 packaging mechanisms [28,29]. It is believed that the T4 phage may not need strictly defined *pac*-sites to initiate DNA packaging due to the characteristics of infection development in the host cell [28,29,30]. The lack of specificity implies that the subunit would interact with any DNA sequence, including sequences of bacterial DNA [25]. Whether such a non-specific binding occurs within the infected cell is another question. Most probably not, and there is a reason for that. During infection, endonucleases expressed by the genome of phage T4 will cut the host DNA into fragments [31]. The phage DNA is protected from the action of endonucleases, as it contains non-canonical bases and their modifications [30]. Thus, with no host DNA left in the cell, and with all of the phage DNA packed, the frequency of transduction will be greatly reduced. This is confirmed experimentally, by obtaining transducing mutants of phage T4 [23,32,33,34,35,36,37,38,39,40]. These works demonstrate that mutations in the genes associated with non-canonical DNA bases and their metabolism, as well as the *ndd* gene, affect the frequency of plasmid transduction. In other words, the frequency of generalized transduction in T4-related phages depends on the presence of non-canonical DNA bases and some proteins directly or indirectly associated with their metabolism.

Phages RB43 and RB49 lack most of the genes associated with non-canonical bases, but they do have genes encoding small terminase subunits (TerS). Moreover, these genes are a part of their core genome [30]. Given that all small terminase subunits are homologous to gp16 of phage T4, one can suppose that they are also non-specific in their interaction with DNA and do not have strictly defined *pac* sites for the recognition of phage DNA. That would explain the ability of phages RB43, RB49, and RB42 to perform generalized transduction with high frequency.

T4-related phages meet the existing safety requirements and, according to them, are well suited for phage therapy. Accordingly, the idea of this paper was to further refine the process of their selection, scrutinizing TerS-containing T4-related phages on their ability to transfer genetic material. On the basis of the aforesaid, we defined the selection criterion as the presence or absence of genes associated with non-canonical bases and, using clustering algorithms, have analyzed the genomes of TerS-containing T4-related phages available in GenBank. As a result, the phages have been divided into groups of “probable low transduction” and “probable high transduction” phages (correspondingly, safer and less safe for the therapy from the viewpoint of their involvement in horizontal gene transfer). It is worth noting that, to date, there is no consensus on what should be considered a “low” and “high” transduction frequency. In different works, the definitions of these terms vary. In general, based on the data for transducing T4 mutants, a frequency of generalized transduction in the range of 10^−1^–10^−7^ can be considered high for T4-related phages, and a frequency less than 10^−8^ can be considered low [32]. We chose the upper limit for low transduction frequencies on the basis of the available phage-T4 data. In the studies on phage T4 transduction, no transductant colonies were obtained [22,33,34,35], and the authors specified the phage concentrations used in the experiments (10^8^ and 10^9^ PFU) as the upper limits for the range of low transduction frequencies. No transduction experiments were conducted for higher concentrations of phage T4. Correspondingly, we chose the highest value out of those obtained experimentally: <10^−8^.

## 2. Materials and Methods

### 2.1. Selection of Genomes

From GenBank CDS translations + PDB + SwissProt + PIR + PRF (excluding environmental samples from WGS projects), we searched the taxa of *Caudoviricetes* (taxid: 2731619) and unclassified *Caudoviricetes* (taxid: 2788787) for the homologs of gp16 of phage T4 (terminase small subunit Escherichia phage T4, accession number: NP_049775) using PSI-BLAST algorithms. The search was performed in four iterations (further iterations did not yield any statistically significant results). The parameter of e-value, at which the sequence was considered homologous, was less than 0.005. The selected sequences were further screened to exclude phage strains that do not infect *E. coli.* The complete genomes of the TerS-containing T4-related coliphages were taken from the NCBI nucleotide collection. After checking them for the presence of incompletely specific bases (represented in the genomic sequences as N, R, M, etc.), the genomes that passed the check were used for cluster analysis. It is worth noting that a number of phages are represented by more than one genome (e.g., Enterobacteria phage RB43 isolate RB43 GVA, Escherichia_phage_RB43), which is due to the fact that the same phage could be sequenced by different scientists, which is especially typical for phages used as model objects. It was decided to disregard such genomes, as the methods of assembling such genomes could differ, which in turn could affect the final genomic sequence. Additionally, the genomes can represent different isolates.

### 2.2. Annotation of Genomes

To avoid problems associated with the use of different annotation methods for the selected phage genomes from the NCBI Nucleotide Collection, we re-annotated all genomes included in the final list using Pharokka v.1.7.3 [41], with standard parameters.

### 2.3. Cluster Analysis

The homologous amino acid sequences of the selected genomes were analyzed using PIRATE v. 1.0.5 [42], with the following parameters: *-s* (identity percentage thresholds), “20,25,30,35,40”; *-- cd-step* (CD-hit step size), 1; *-- cd-low* (minimum CD-hit identity percentage), 90; *-e* (e-value), 1 × 10^−4^; and -f (mcl inflation value), 1.4. Other parameters remained at their default values.

### 2.4. Multiple Alignment and Phylogenetic Analysis of TerS Sequences

The multiple alignment of TerS sequences, which were taken from the resulting homologous cluster, was performed on the basis of the MUSCLE algorithm using Unipro UGENE v.50 [43] with standard parameters. The phylogenetic analysis of the aligned TerS homologs was conducted using IQtree v.2.3.6 [44]; the branches were supported with ultrafast bootstrap approximation (UFBoot; [45,46] and SH-like approximate likelihood ratio test [47]). For each branch support, 1000 bootstrap replicates were used.

### 2.5. Visualization of Bipartite Networks

On the basis of a PIRATE-generated matrix of the presence/absence of homologous clusters, we constructed a table of node interactions in a bipartite gene-exchange network, with the first set of nodes representing genomes and the second representing clusters of homologous sequences. The bipartite network was visualized using Cytoscape v. 3.10.2 [48] with the following parameters: *layout algorithm*, prefuse force directed layout; *number of iterations*, 10000; *default spring length*, 100; and default node mass, 8. Other parameters were set at their default values. For the collapsed network, the default spring length and default node mass parameters were changed to 200 and 20, respectively.

**Figure 1:**
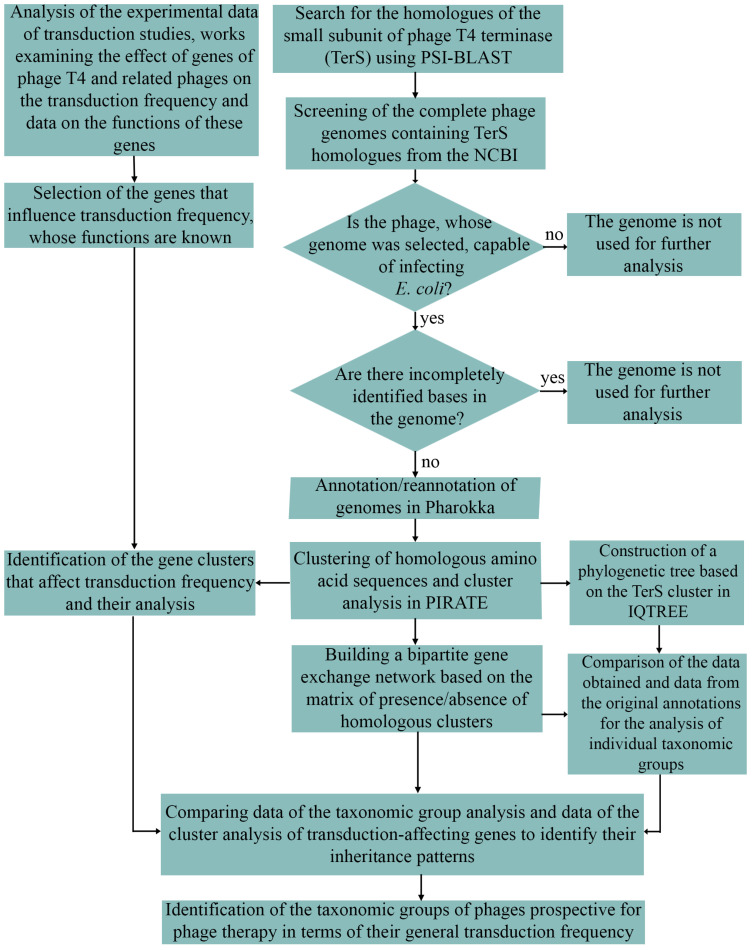
Flowchart illustrating a bioinformatics approach and search strategy to identify T4-related phages with reduced transduction frequency for therapy.

Our approach is summarized as a flowchart in Figure 1.

## 3. Results

### 3.1. Selection of Genes Affecting Transduction Frequency in T4-Related Phages for Cluster Analysis

T4-related bacteriophages, which carry non-canonical bases in their DNA, as well as genes regulating their metabolism, seem to transduce genetic material with a rather low frequency. For example, wild-type phage T4 was shown to transfer *E. coli* genes involved in the metabolism of sugars and amino acids (*ArgG*, *Thr*, *Ara* etc.) with a frequency less than 10^−8^–10^−9^, and no colonies of transductant bacteria were obtained in early experiments [33,34].

**Figure 2:**
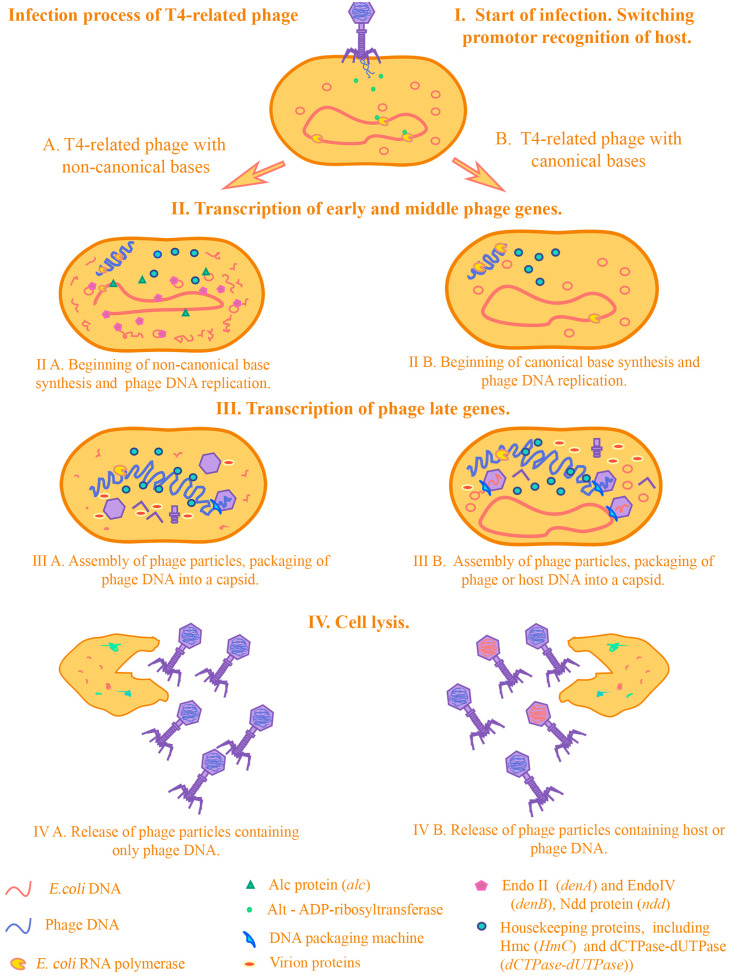
The process of infection with T4-related phage genes that can reduce/increase their transduction frequency. The legend in parentheses indicates the genes of T4-related phages that we used in our analysis. A—The process of infection with T4-related phage genes that can reduce their transduction frequency. B—The process of infection with a T4-related phage without genes that can reduce their transduction frequency.

We have conducted a study of the literature data concerning experiments on transducing mutants of T4. Based on these data, we have selected genetic determinants that can serve as a marker for decreased/increased transduction frequency in T4-related bacteriophages. In the text below, we will try to explain why the genes we have selected serve as a marker for decreased transduction frequency in T4-related coliphages using bacteriophage T4 as an example. In Figure 2 we demonstrate how transduction occurs in T4-related phages in the presence/absence of the genes we selected.

When infected with a T4 phage, the fact of gene transfer by transduction is practically excluded for several reasons. First of all, this is due to the infectious process of the T4 phage. At the beginning of infection, some phage proteins redirect host RNA polymerases to their phage promoters due to proteins that perform ADP-ribosylation of the alpha subunits of the RNA polymerase of *E.coli*.

One of these proteins is the Alt protein, which enters the cell directly from the phage capsid [49]. Then, the expression of early genes of the phage begins. Among these is the Alc protein, which affects transcription and indirectly the replication processes of the host DNA. Alc functions as a site-specific transcription termination factor on cytosine-containing DNA [50]. It was shown on the transduction of the pBR322 plasmid that mutations in the *alc* gene have a strong effect on the transduction frequencies of plasmids by transducing T4 mutants. In particular, it was shown that transduction of pBR322 can occur by the T4 phage with mutations in both the *denB* and *alc* genes, and not only in *denB* (transduction frequency <10^−7^). The authors of the study suggest that this is due to the indirect effect of Alc on plasmid replication: due to blocking the synthesis of replication initiator proteins, the synthesis of RNA primers necessary for the initiation of plasmid DNA replication is also blocked [51]. Thus, proteins that affect the processes of transcription and translation from *E.coli* DNA during infection can have a strong effect on the transduction frequency. At the moment, there are experimental data only on one of these proteins—Alc. It can be assumed that the products of the genes *modA*, *modB*, and *alt*, which are related to ADP-ribosyltransferases, can also affect the change in transduction frequencies, by analogy with Alc. However, we did not include these in the analysis due to the lack of experimental data on transducing mutants for the above genes. The next step in the infection process is the destruction of the host DNA. This is a necessary step for several reasons—bacteriophage T4 is a large phage and the cell needs space to reproduce phage particles. Moreover, free dNTPs are needed to synthesize phage DNA. Phage T4 has several endonucleases—including Endo II (DenA) and EndoIV (DenB)—which hydrolyze the cytosine DNA of *E. coli*, but not the phage DNA, which contains modified 5′-hydroxymethylcytosine bases. An important role is played by the Ndd protein, which is incredibly toxic to the cell. Ndd destroys the structure of the *E. coli* chromosome, completely blocking the repair and replication processes in the host DNA [52]. Correspondingly, during infection, the enzymes will hydrolyze *E. coli* DNA and leave phage DNA intact [53].

Destruction of the host DNA prevents it from entering the forming phage particles, thereby reducing the frequency of transduction. Numerous experimental data on transducing T4 mutants in these genes confirm this. There are conflicting data on which of the endonucleases, DenB or DenA, has the greatest impact on the destruction of plasmid DNA, and therefore the possibility of transduction. This contradiction was resolved in the article by H E Selick et al., where it was confirmed that DenB is the main nuclease that participates in the destruction of plasmid DNA [40]. The contribution of DenA to the degradation of chromosomal and plasmid DNA is no less significant, as was shown in research by Mattson et al. [54].

Next, non-canonical bases included in the phage DNA are synthesized and modified. The low frequency of transduction by T4-type bacteriophages may be a result of the presence of 5-hydroxymethylcytosine (HmC) in their genome. HmC, a derivative of cytosine, replaces the latter in the phage DNA. In many ways, it is the presence of non-canonical bases in the T4 phage that contributes to limitations in participation in horizontal gene transfer by transduction. It was demonstrated that amber mutations in the genes of the enzymes responsible for HmC synthesis (genes *42* and *56*, encoding HmC transferase and dCTPase-dUTPase, respectively), as well as deletion of the region containing *denB*, a gene of endonuclease IV, dramatically raised the frequency of genetic transfer. For *E. coli* genes, the frequency increased to 10^−5^–10^−7^, i.e., 100–1000 fold [34] and for plasmids (in particular, pBR322), to 10^−2^–10^−3^. Their presence in itself does not affect whether this process will occur, however, due to the genes associated with them and their effect on the development of infection, particularly those considered above, the possibility of horizontal transfer is greatly reduced. This was noted in several experimental articles on the evolution of T4-related phages [30,55].

The remaining DNA in the cell is packaged into forming phage particles using a packaging machine. The main function of packaging DNA into capsids is performed by terminases—large and small subunits (TerL and TerS). As noted earlier, a strictly specific region of recognition by terminases has not been identified, presumably, these can be regions in the region of genes 16 and 19. It is assumed that recognition is due to the conformation of phage DNA (during packaging, phage DNA is in a state of multiple concatemers formed due to the process of replicative recombination) at the time of packaging [26,27,56,57]. Work on the transduction of plasmids by transducing T4 mutants confirms this [32,58,59]. Thus, whether transduction will occur or not depends on the genes that directly affect the processes of transcription, translation, replication of chromosomal and plasmid DNA of *E. coli*. These genes in some studies were combined under the name host alteration/shutoff [49].

As experimental data on the effect on transduction frequency were determined only for genes *denB*, *denA*, *ndd*, *alc*, *hmc* and *dCTPases-dUTPase* we decided to analyze them. The information about the genes of T4-related phages that can influence the frequency of transduction is summarized in Table 1.

In experiments on the effect of the T4 phage’s own genetic determinants on the transduction frequency [34], a set of mutants was used, as follows: T4GT7 [33], T4GT4, NB5060 [36,37], NB3157 [36,37], 1272 [36,37], SaΔ4 [38], SaΔ5 [38], SaΔ9 [38], 184, 196, E51, C86 and their recombinants. Phage T4GT7 (*amC87* and *amE51 Δ(rIIB-52) alc*) is one of the widely used mutants for studying various aspects of molecular biology today. Due to this, its genomic sequence is available and the differences between this genomic sequence and the T4 genome are known [60]. This phage includes amber mutations in the genes of hydroxymethylcytosine transferase (*amC87* or *am42*), dCTPase-dUTPase (*amE51* or *am56*), deletion NB5060 of region D (see below) and spontaneous mutation of the *alc* gene. Phages NB5060, NB3157, 1272 (by the name of the deletion) are deletion mutants of region D with different lengths of the deletion fragment [36]. Region D is a fragment of the T4 phage genome between the *rIIB* and *52* genes (DNA topisomerase II medium subunit gene) [36,38]. This region, of the genes considered in this work, includes *denB*, *ndd*. Additionally, like the mutants described in the previous sentence, SaΔ4, SaΔ5, SaΔ9 are deletion mutants of region D (they have deletions different from the previous phages) [38]. SaΔ were obtained independently from mutants NB5060, NB3157, and 1272, which is worth noting.

The following mutants are also presented in [34]: T4GT4 (*am56 Δ(rIIB-52) alc*), 184 (deletion of the *rIIA* gene), 196 (deletion of the *rIIB* gene), E51 (*am56*), C87 (*am42*). To clarify the influence of the genetic determinants of region D, the *hydroxymethylcytosine transferase*, *dCTPase-dUTPase*, and *alc* genes, the transduction frequencies of the chromosomal marker *Arg* (necessary for the growth of the arginine auxotrophic recipient strain of *E. coli*) by these strains were compared. In addition, recombinants of these strains were used to obtain a wide range of mutation combinations for different genes. This made it possible to identify the influence of individual genes on transduction by comparing the frequencies of “overlapping” mutations. In addition, recombinants with similar mutations were obtained, but their parent strains were different, which to some extent allowed us to cut off the influence of factors unrelated to the mutations considered in [34] on transduction. For all transduction experiments with different mutants, the *E. coli* strain QD sup3 (*pro*, *supIII*) was used as a donor, and JC411 (*metB leu his argG lac malA xyl mtl^-^ gal str λ^-^*) was used as a recipient [23,34].

Unfortunately, there are no detailed experiments on the effect of DenA on transduction. It has been noted [23] that the T4β-alc mutant (*amN55 (am42)*, *amE51*, *nd28 (denA)*, *rIIH23*, *alc*) [39] has a similar transduction efficiency to phage T4GT7, but no comparison of transduction frequencies was presented. The *rIIH23* deletion contains the *denB* gene [39]. There are also data on the effect of the *denA* gene on the integration of a plasmid with a fragment of phage T4 DNA into the genomic DNA of phage T4 [40]. For this purpose, the frequencies of plasmid integration into DNA of the T4 I/S mutant (*amB262*, *amS29*, *nd28*, *rIIPT8*) and its recombinants with T4 JW15 (*46^-^*, *denA^+^*, *denB^+^*) were compared: I/S *denA^+^*, I/S *denB^+^*, T4 I/S *denA^+^ denB^+^*. Mutation *amB262 (38^–^)* is an amber mutation in the fibril adhesin gene, *amS29 (51^-^)* is an amber mutation in the baseplate hub assembly protein gene, *nd28* is a mutation in the *denA* gene, *rIIPT8* is a deletion in the D region, including the *denB* gene. In the experiments on measuring the integration frequency, the *E. coli* strains AB1 (nonsuppressing) or MCS1 (*supD*) were used as donors, and BE (nonsuppressing) and CR63 (*supD*) as recipients. The plasmids used were pBSE0f+ and pBS4f+, which contained the *supF* gene, as well as a fragment of the *frd* gene of the T4 phage (dihydrofolate reductase). The plasmid integration frequency was calculated as the ratio of the number of phage plaques formed on the BE strain to the number of plaques formed on the CR63 strain. As noted in [40], DenA reduces the plasmid integration frequency by approximately 2–5 fold. Taking this into account, as well as the function of this protein, it can be assumed that this protein is able to reduce the frequency of plasmid transduction due to endonuclease activity. Thus, the absence/mutation of this gene increases the possibility of preserving the plasmid in a cell infected with a phage, thereby increasing the likelihood of plasmid DNA entering the capsid.

In order to reduce the possible influence of secondary mutations in case of the mutations described above, as well as the effect of mutations in other genes, the strains used in [36,37,38] were either independently isolated mutants by the same genes/regions or recombinants whose parental strains were independent. In addition, the use of mutants and recombinants with cross- or different mutations made it possible to determine or specify the influence of target genes. This approach apparently allowed the authors to substantially reduce the influence of secondary mutations on the experimental results, although the problem might not have been completely solved. The approaches used for mapping and testing of strains with mutations in the D region (RNA hybridization-competition, genetic crosses and electron microscopy of DNA heteroduplex molecules [36,37,38]) also helped to avoid the influence of secondary mutations.

Thus, mutations in the T4-phage genes responsible for the synthesis of non-canonical bases and degradation of host DNA or deletion of these genes can significantly increase the transduction frequencies of plasmids and some *E. coli* genes. As a result of their inactivation, host DNA remains in the cell during infection, leading to its misrecognition by TerS and packaging initiation into the capsid.

Based on these experimental data and information about TerS functions, we screened *Caudoviricetes* for TerS homologs, downloaded genomes of the selected coliphages from the NCBI database collection and checked them for the presence of the above-listed genes, assuming that their absence would be an indicator of an elevated frequency of genetic transduction.

### 3.2. Selection of Genomes of T4-Related Phage with TerS and Their Cluster Analysis

The number of TerS-containing coliphages, whose genomes were downloaded, totaled 431; these belonged to the families of *Straboviridae* and *Ackermannviridae*.

A total of 122,198 open reading frames obtained from genome annotations were further analyzed, and their amino acid sequences were arranged into 1,681 homologous clusters. The number of homologous clusters versus the percentage of genomes is shown in Table 2, and the occurrence of transduction-reducing genes in these genomes is given in Table 3. Information on all bacteriophage genomes used in the analysis is presented in Table S1: List of phages. The table presents phage numbers, their name, taxonomy, data on the presence or absence of genes *dCTPases-dUTPase*, *Hmc*, *denB*, *denA*, *ndd*, *alc* and assumptions about the frequency of generalized transduction.

Table S2: “Homologs of genes defined as components of the softcore genome of the viruses” presents genes that occur in 95–100% of genomes (such genes represent the softcore genome). The softcore genome is more useful than considering only the core genome, due to possible errors that occur during genome assembly/annotation. The largest number of softcore genome genes was found among the homologs involved in morphogenesis, which is not surprising given the large number of virion components in these phages. The obtained data on the softcore genome components are similar to those previously obtained in the study of the pan-genome of T4-related bacteriophages in other studies [55]. In this case, a larger number of softcore genome components can be noted, which is probably due to the fact that the genomes of phages infecting E. coli were used, as a result of which the softcore genome could include genes associated with adaptation to these bacteria. The presence of a small number of core genes (15) may in some cases be due to the use of phage family *Ackermannviridae* genomes in clustering, which are evolutionarily the most distant from the other selected phages. It is worth noting, however, that the most conserved genes, such as the genes for the major capsid protein and the large subunit of terminase, are defined as core genes. Interestingly, among the genes involved in auxiliary metabolism were genes that affect the frequency of transduction: *denA* and *dCTPase/dUTPase*.

**Figure 3:**
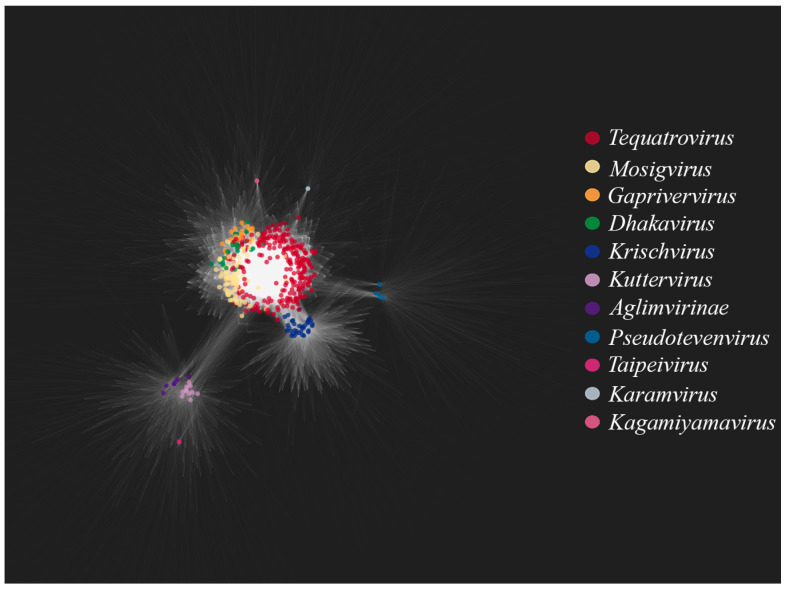
A bipartite network of gene share constructed for the TerS-containing phages. Colored circles indicate nodes of phage genomes belonging to *Straboviridae* or *Ackermannviridae* genus and subfamilies. For clarity, the nodes of homologous clusters are not marked.

Figure 3 shows a bipartite gene exchange network for the TerS-containing phages. One can see that the genera *Tequatrovirus*, *Mosigvirus*, *Gaprivervirus* and *Dhakavirus* form a dense united subnetwork. This is not surprising since they belong to the same subfamily, *Tevenvirinae.* The closest to the *Tevenvirinae* subnetwork are dense subnetworks of the *Krischvirus* and *Pseudotevenvirus* genera and the nodes of individual representatives of *Kagamiyamavirus* and *Karamvirus*; all of these genera (along with *Tevenvirinae*) belong to the family *Straboviridae*. Located separately is a dense subnetwork of the *Kuttervirus* and *Taipeivirus* genus and the subfamily *Aglimvirinae*, which are taxons of the family *Ackermannviridae*. The network, therefore, reflects modern views on the evolution and classification of viruses. The bipartite network reflects the modern view of the evolution and classification of viruses, as individual taxonomic groups (families, subfamilies, genera) visually form dense subnetworks.

**Figure 4:**
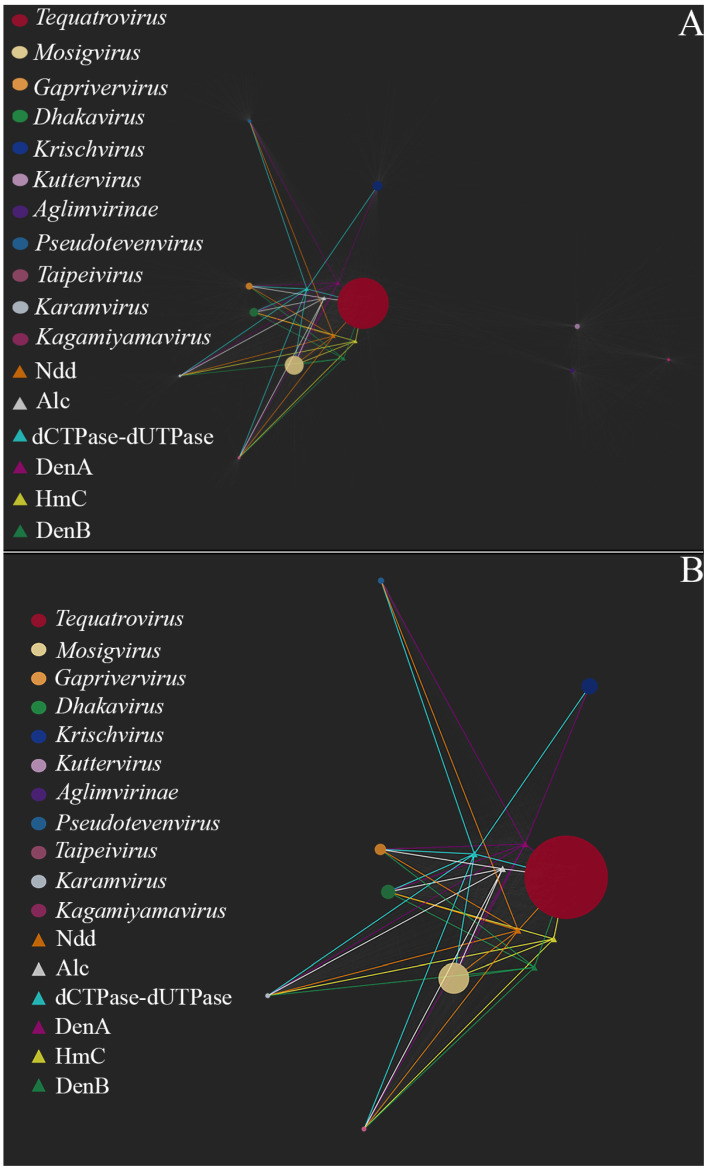
A bipartite network of gene share among TerS-containing phages, which reflects relationships between genomes and clusters of homologous genes associated with decreased transduction frequency. (**A**) A zoomed-out view of the entire network, with the separate phage genome nodes collapsed into the united nodes of the genus that the phages belong to. The nodes are drawn as circles whose size is proportional to the number of phages analyzed. The nodes of gene clusters associated with decreased transduction frequency are drawn as triangles, and their relationships to the circular genome nodes are depicted as connecting lines drawn in the same color as triangles. (**B**) A close view of the fragment of the network showing the nodes of gene clusters associated with decrease transduction frequency and their relationships to the genome nodes.

Figure 4 shows relationships between the nodes of homologous gene clusters associated with decreased transduction frequency and the nodes of the analyzed genomes. As can be seen in Figure 4, phages of the genus *Tequatrovirus*, *Mosigvirus*, *Dhakavirus*, *Karamvirus* and *Kagamiyamavirus* have the largest set of genes associated with decreased transduction frequency (namely *denB*, *denA*, *ndd*, *HmC*, *dCTPase- dUTpase* and *alc*). The phages of these genus have non-canonical bases in their DNA (glucosylated or arabinosylated derivatives of common bases, including HmC, etc.) and, so far, there have been no reports that the frequency of transduction by these phages exceeds 10^−8^–10^−9^. Furthermore, there are experimental data indicating that phages from the genus *Mosigvirus* and *Tequatrovirus* are capable of infecting pathogenic strains *of E. coli*. This was confirmed in both in vitro tests and in vivo experiments on animals. Based on this, we can say that, out of all the phages considered, the representatives of the above-mentioned genus of the *Tevenvirinae* subfamily are the most promising candidates for phage therapy.

An interesting case, which is worth mentioning, is the genus *Gaprivervirus*. Phages of this genus have homologs of the transduction-reducing genes, with one exception: they lack the gene of HmC transferase. Overall, with the set of genes they carry, *Gaprivervirus* phages would not be able to replicate without non-canonical bases in their DNA. These viruses may have a non-canonical base which is currently unknown. One can assume that *Gaprivervirus* phages should be low transducing; however, this is still to be confirmed, and further studies are needed to estimate their potential for therapy.

As for *Ackermannviridae*, no homologs of the transduction-reducing genes are detected in the phages of this family, which may indicate them to be high-transducing. This conclusion is confirmed by the data on plasmid transfer by *Ackermannviridae* representatives [61,62]. More specifically, it has been shown that ViI-type phages (in particular, kuttervirus ViI) are capable of transducing plasmids and chromosomal elements with a rather high frequency of 10^−5^–10^−6^, and the researchers suppose that a high frequency of generalized transduction is characteristic of all ViI-type phages [61]. According to the modern classification, ViI-type phages include the genus *Kuttervirus*, which represents the majority of *Ackermannviridae* phages selected for our analysis [62]. What is interesting about this particular genus is that its phages carry homologs of the genes synthesizing 5-(2-aminoethoxy) methyluridine [63], a hypermodified non-canonical base. Like HmC, it can presumably reduce the frequency of transduction during DNA packing, though further studies are needed to confirm this.

Phages of the genus *Krishvirus* (e.g., the transducing bacteriophage RB49) and phages of the genus *Pseudotevenvirus* (e.g., the transducing phages RB43 and RB42) lack the genes of HmC transferase and endonuclease IV. As we know from the study of T4 mutant E51-NB5060 [34], discussed above, switching off these genes has no effect on the ability of phages to transduce genetic material; moreover, the frequency of transduction even increases, to experimentally detectable levels. According to our own experimental data, phage RB49 transfers plasmids with a frequency of 10^−4^–10^−6^, and, although there are no data on the frequency of transduction of chromosomal genes, it is usually lower when compared with the frequency of plasmid DNA transfer. Presumably, it should be in the same range as in T4 mutant E51-NB5060 [34].

It is worth noting that, unlike *Krishvirus* phages, phages of the genus *Pseudotevenvirus* have *ndd*. It can, therefore, be assumed that the frequency of plasmid transduction in RB 43 is somewhat lower. However, according to our experimental data, it is in the range of 10^−3^–10^−6^. Perhaps, the deletion *of ndd* itself does not increase the frequency of plasmid transduction as much as the deletion of *denB*. In the study by Takahashi [32], the mutant phage T4 C(+), carrying mutations in four genes (*42*, *56*, *denB* and *alc*), but not *ndd*, was shown to have a transduction frequency of pBR322 which was five times lower than that of the mutant T4dC (NB5060) with the deleted region *rII-ac.*

It should be noted that transduction frequencies for *Krischvirus* and *Pseudotevenvirus* are in the range of 10^−5^–10^−7^ [22,24]. As a rule, the concentrations of phages used in therapy are rather high, from 10^7^ and above. If the bacterial strain used for phage cultivation is not chosen carefully and has plasmids, the preparation will probably contain transducing particles. Even if the phage preparation is obtained from a “safe” bacterial strain, without plasmids and virulent factors, its administration in large doses can result in high and persistent titers of phage particles in the organism [64]. Correspondingly, the probability of the host DNA being packed into the capsid during the development of phage infection will be high.

Summing up the results of our analysis, we would recommend avoid using T4-related phages from the genus *Krishvirus* and *Pseudotevenvirus* of the family *Straboviridae* and phages of the taxa *Kuttervirus*, *Taipeivirus* and *Aglimvirinae* of the family *Ackermannviridae* for therapeutic purposes. At least, they should be used with great caution for the treatment of *E. coli* infections. As for the phages of the genus *Gaprivervirus*, we still cannot give recommendations about their therapeutic use, since it is unclear if their DNA contains noncanonical bases.

The analysis of gene clusters associated with the decreased frequency of transduction revealed that, in the family *Straboviridae*, these genes were shared among representatives of the same genus. The exception was Escherichia phage EC.W15-4 (accession number: PP500713.1), which, in contrast to other representatives of *Tequatrovirus*, lacked the *alc* gene. Most probably, this was just an artefact, resulting from the incorrect identification of the reading frame of the gene (which could happen if, upon genome assembly, one part of the gene was placed at the beginning and another, at the end of the genome).

Data on the presence/absence of the homologs of genes affecting the transduction frequency for individual taxonomic groups and the possible presence of a high transduction frequency in representatives of these groups are presented in Table 4.

### 3.3. Multiple Alignment and Phylogenetic Analysis of TerS Sequences

On the basis of clustering data, we built a phylogenetic tree for the TerS of T4-related coliphages. All of the TerS sequences analyzed were homologous to TerS of phage T4 (gp16). The result of the phylogenetic analysis is presented in Figure S1: Phylogenetic tree of TerS. Ultimately, sequences from the same taxons formed separate clades. This is not surprising, as terminases are conservative within each genus, and can be used as identifiers of the latter, just like the major capsid protein.

One can see that there are two large clades on the tree: first, represented by the family *Ackermannviridae* and second, by the family *Straboviridae*. According to the homology analysis and amino acid sequence alignment data (File S1: alignment of TerS), small terminase subunit of *Ackermannviridae* coliphages have little in common with gp16 of phage T4. For example, the small terminase subunit of AV101 is 25.21% identical to that of the T4 phage, with a coverage of 65%. However, given that *Ackermannviridae* phages have a headful packing mechanism [61,65] and can transfer plasmids with a high frequency [61], we decided to include them in the analysis.

Within the *Ackermannviridae* clade, the sequences further branch into subclades corresponding to the phage genera: *Kuttervirus*, *Aglimvirinae* and, as a separate branch represented by a single sequence, *Taipeivirus*.

The small terminase clade of the family *Strabovivridae* is divided into several large subclades formed by sequences from the genus *Pseudotevenvirus*, *Tequatrovirus*, *Mosigvirus*, *Dhakavirus*, *Gaprivervirus*, and *Krishvirus.* The exceptions are sequences from the genus *Karamvirus* and *Kagamiyamavirus*, as they each have only one representative. It is worth noting that TerS of *Pseudotevenvirus* are quite distant from those of other *Strabovivridae* phages. Interestingly, there are experimental data indicating that *Pseudotevenvirus* coliphages can transfer plasmid DNA with a high frequency. Perhaps there is a connection here, and the mechanism of action of *Pseudotevenvirus* terminases differs from that of other *Straboviridae* terminases, affecting the frequency of plasmid transduction by the coliphages of this group.

## 4. Discussion

It is worth noting that our bioinformatic approach has a number of limitations that we would like to discuss below. In selecting phages, data from experiments on transducing mutants of the T4 phage was obtained, and those genetic determinants known to affect the transduction frequency were selected. This approach was validated through experimental studies. However, it is noteworthy that the *Krischvirus* and *Pseudotevenvirus* phages, despite their T4-related characteristics, exhibit significant differences from the T4 phage [30]. Although the biology of these viruses is well understood, there is a lack of research on their ability to transduce plasmids at high frequency. The emergence of new experimental data in this area will reveal more details of this process and improve our approach to the selection of safe phages for high-frequency transduction.

It is also worth noting that our approach affects only the genetic characteristics of the phage itself, but not the host bacteria. The focus remains exclusively on generalized transduction, while the success of horizontal transfer is influenced by the genetic characteristics of the bacterium. The presence of prophages, plasmids, and restriction–modification systems within the bacterium can influence horizontal transfer [66,67,68]. The emergence of new experimental data on the transduction of different plasmids by the same T4-related phage into various *E. coli* strains may provide further insights into these questions. It is also important to highlight recombination processes that can occur if the host bacterium contains homologs of the genes of the phages used [30]. Furthermore, there is a possibility that there may be other bacteriophages in the patient’s gastrointestinal tract, and the interaction of therapeutic phages from the cocktail with them is currently poorly understood [9]. The obtaining of experimental data on the interaction of therapeutic phages with the intestinal microbiota has the potential to improve approaches to the selection of agents for therapy, including the one given in this article.

At this stage, we can say that our approach is largely based on the presence/absence of T4-related coliphage genes associated with non-canonical bases. This choice is also due to the biology of phages with non-canonical bases, the presence of which not only protects phages from the defense systems against the foreign DNA of host bacteria, but also possibly helps them better preserve their genome during evolution [55]. Phages with non-canonical bases, due to the peculiarities of infection development in the cell and the presence of a pool of genes responsible for the synthesis of non-canonical bases and their metabolism, reduce the risks of involving the genetic material of host bacteria in recombination processes [30,55]. This is a fairly complex process. However, the following possible influence of the genes under consideration on transduction can be assumed.

In experiments on T4-transducing mutants, it was demonstrated that the genes responsible for the degradation of cytosine DNA in the host bacterium play a crucial role in their ability for generalized transduction [23,32,33,34,35,36,37,38,39,40]. During infection, the action of the DenA and DenB endonucleases, as well as the Ndd protein of the T4 phage, results in the complete degradation of the host cytosine DNA, a process which releases the nucleotides necessary for the synthesis of phage DNA. A notable increase in transduction frequency (10^−8^) was observed in mutants (NB5060 and NB3157) that had a deleted region containing *denB* and *ndd* [34]. However, the removal of these genes made it possible to obtain transductant colonies carrying the genes of the donor bacterium compared with the wild type T4. Consequently, it can be deduced that mutations or the absence of genes associated with host DNA degradation during infection can have a significant role in the ability of the lytic phage to generalized transduction. This phenomenon has been previously observed in the study of the lytic phages *E. coli* SUSP1 and SUSP2 [69], where the absence of the homologs of *denA*, *denB* and *ndd* resulted in the high-frequency transduction of plasmids. The phage DNA is protected from the action of its own nucleases due to the presence of 5′-hydroxymethylcytosine (product of gene *42*), which has an additional modification in the form of alpha or beta-glucosylation. Mutations in genes *42* and *56* in the T4 phage do not lead to an increase in the transduction frequency [34]. However, mutants with genes *42* and *56* and with the deletion of endonuclease genes showed the highest transduction frequencies [34]. This phenomenon may be attributed to the reduced efficiency of the infection process, as the phage DNA is now susceptible to degradation by the cell’s defense systems (restriction–modification systems, CRISPR–Cas systems, etc.). Furthermore, evidence suggests that, in cytosine mutants of T4, DNA transcription may be disrupted at later stages [50]. The presence of non-canonical bases in the genome provides protection not only from its own DNA degradation systems with canonical bases, but also from the cell’s defense systems against foreign DNA. If phage endonucleases limit the possibility of foreign DNA entering the capsid, then non-canonical bases help maintain the integrity of their own DNA and thereby also ensure that the capsid is filled primarily with phage DNA, rather than host DNA, reducing the transduction frequency. Thus, a T4-related phage without non-canonical bases and genes associated with them has a greater chance that foreign DNA can enter the phage capsid during infection.

All of these genes are included in the softcore genome of T4-related phages. In this article, we did not consider genes from the core genome and their effect on transduction. Genes from the core genome of T4-related phages are responsible for the replication, recombination, transcription, and translation of phage DNA. Their deletions and mutations will disrupt phage metabolism and affect not only such a process as transduction, but also the vital activity and viability of the phage particles themselves. Of course, genes responsible for replication by recombination, such as *uvsX*, *uvsY*, *46*, *47*, *39*, *52*, *60* and others, can also be considered as markers of high or low transduction frequency. It has been shown that the replication–recombination process in T4 can enable the packaging of plasmid DNA into the phage capsid under several conditions [58,59,70]. In particular, this plasmid contains an insert of phage DNA, which makes homologous recombination possible, and T4 contains mutations that prevent host DNA destruction (in particular, in the *denB*, *denA*, and *ndd* genes). In another case, the plasmid, in addition to the homologous region, may contain the T4 replication origin. The possible influence of homing endonucleases on this process has also been noted. Thus, even in the case of the transduction of plasmids with a homologous region of the phage by a T4-related phage, it is necessary for the phage to have mutations in the genes responsible for the degradation of the host DNA. Thus, the transduction markers we have chosen seem to most fully reflect the processes of the possibility of high- or low-frequency transduction by T4-related phage.

In addition to the genes mentioned above, other proteins associated with non-canonical bases may also affect transduction frequencies. For instance, the Alc has been demonstrated to play a role in this process. This protein is involved in host transcription inhibition following *E. coli* infection, and its absence may therefore affect the survival of host DNA in the cell and its involvement in packaging into phage particles [34,49]. This is confirmed by an increase in transduction frequencies associated with a mutation in this gene. It is hypothesized that other proteins associated with host alteration/shutoff or transcriptional regulation may also affect transduction frequencies. For example, these may be proteins encoded by *alt*, *modA*, and *modB*. It is acknowledged that there may be other genes that may affect transduction frequencies. We did not consider the effects of *rII* on transduction in this article because the function of these genes is not entirely clear, although, according to experimental data, the effect of these genes on transduction may be significant. Further experimentation is necessary to generate transducing mutants of T4 and to study the generalized transduction of *Krischvirus* and *Pseudotevenvirus* phage plasmids. This will provide further insight into the contribution of T4-related phages to horizontal gene transfer.

It should be noted that *Krischvirus* and *Pseudotevenvirus* phages carry significant number of genes whose function is not known. There is no guarantee that some of these genes affect or do not affect transduction. Given what we know about the *Krischvirus* and *Pseudotevenvirus* phages, we can only assume that their capability to transduce is related to specifics of their vital activity: in particular, to the mechanism of genome packing into the capsid. This is similar to that observed in *Tequatrovirus*, but is less specific towards their own DNA: *Krischvirus* and *Pseudotevenvirus* phages lack the genes associated with noncanonical bases, and, in particular, endonucleases, which would cut the host DNA.

Therefore, the theoretical foundation of our approach, based on the current understanding of T4-related phages at this stage of phage research, is, in our opinion, well supported.

The bioinformatics approach can be widely used for a variety of purposes in phage therapy. For example, genetically engineered phages were included in a cocktail used to treat a 15-year-old patient from *Mycobacterium abscessus* (two out of three phages used in the cocktail had their repressor genes removed, resulting in the switch of their life cycle from lysogenic to lytic [71]). The development of effective derivatives of the lytic phage for this cocktail became possible due to the sequencing, annotation and comparison of phage genomes from a database of 1800 sequenced phages. Thanks to this, it was possible to determine which changes or deletions of which genetic determinants can increase the efficiency of the phage infection of the *Mycobacterium abscessus* [71]. Thus, thanks to various bioinformatics tools, it is possible to select a phage that is effective for therapy.

Our bioinformatics approach can be used as a first step in selecting a suitable object for phage therapy. We propose to use the genetic determinants we selected, in particular the *hmC* and *denB* genes, as markers for selecting T4-related coliphages suitable for therapy. Given that the sets of transduction-affecting genes are genus-specific, the selection can also be conducted on a group basis, e.g., by selecting specific primers for the capsid proteins gp23 of a certain genus of *Tevenvirinae* phages.

Thus, the practical side of the issue arising from our method is the development of specific PCR tests for each of the presented phage genus from the *Tevenvirinae* subfamily. This will allow the selection of phages that are safe for therapy at the stage of their purification and isolation from natural sources and will significantly reduce the time need to select a safe phage. Clearly, bacteriophages selected according to this principle should be subjected to experimental testing for plasmid transduction in vitro to ensure its safety. The principles we have proposed for phage selection can be extrapolated to other T4-related bacteriophages with non-canonical bases, including yet undiscovered coliphage taxa. Experimental data on plasmid transduction by coliphages of the *Dhakavirus*, *Gaprivervirus*, *Kagamiyamavirus*, *Karamvirus*, and *Mosigvirus* are needed to fully test the feasibility of our approach. For now, there is no evidence which would suggest the presence of similar mechanisms for decreasing transduction frequency in other phages. At the same time, there are many data on the presence of similar noncanonical bases (hypermodified pyrimidines) in various viruses, although no data are available on the presence of similar endonucleases, degrading the host DNA. This, it would be too early to think about extrapolating the method to all of the known bacteriophages.

Thus, we believe that the proposed approach to the selection of phages with a low transduction frequency can reduce and prevent risks associated primarily with the introduction of unwanted genetic information when creating a phage cocktail. One of the recommendations when obtaining a phage preparation is the absence of any prophages or plasmids in the host bacterium on which the therapeutic phage will be grown. Pathogenic *E. coli* strains often have these, which complicates the task of creating a phage preparation against one strain of pathogenic *E. coli*. Growing the phage on the host strain, and not on a safer one from its host range, has the advantage of the more effective lysis of pathogens. Selecting a coliphage with a low transduction frequency can reduce the risks of any pathogenicity factors finding their way into phage particles, as well as reduce the risk of the transfer of such factors from a pathogenic strain to a commensal strain already located in the patient’s gastrointestinal tract during treatment. Additionally, to obtain a safe phage preparation, it is necessary to conduct whole genome sequencing of the host bacterium on which it is planned to obtain a harvest of therapeutic phage. Furthermore, PCR testing should at least be carried out for the presence of virulence and/or pathogenicity factors of bacteria in the finished phage preparation.

It is currently impossible to completely exclude the possibility of any horizontal gene transfer during phage therapy; however, it is necessary to reduce the risks to a minimum. We believe that our approach to the pre-selection of T4-related coliphages for therapy can help reduce this risk.

## 5. Conclusions

In this work, we present a novel bioinformatics approach to the selection of phages for therapeutic purposes. The approach is based on the assessment of the ability of phages to participate in the horizontal gene transfer and is aimed to minimize the risks of spreading potentially hazardous genes among phages and their bacterial hosts. Summarizing literature data on the transducing mutants of T4 bacteriophage and our knowledge of the biology of T4-type bacteriophages, we have demonstrated how the suggested approach can be used to analyze the group of T4-related coliphages. First, a survey of the available experimental data allowed us to estimate the potential involvement of phages of different taxonomic groups in the horizontal gene transfer by the mechanism of generalized transduction [23,32,33,34,35]. Next, using cluster analysis of the homologous sequences of genomes of TerS-containing T4-related coliphages, we identified the subfamily *Tevenvirinae* as carrying the largest set of genetic determinants that reduce the frequency of transduction (*denB*, *alc*, *denA*, *Hmc*, *ndd* and *dCTPase-dUTPase*). Second to *Tevenvirinae* are coliphages from the genus *Krishvirus* and *Pseudotevenvirus* of the family *Straboviridae,* which have a partial set of the transduction-reducing genes, lacking *denB*, *alc* and *Hmc*. The coliphages of *Kuttervirus*, *Taipeivirus* and *Aglimvirinae* from the family *Ackermannviridae* do not have any homologs of the transduction-reducing genes, suggesting that the frequency of generalized transduction by these phages is relatively high. In accordance with our analysis, we labelled the two groups of phages as the “probable low transduction frequency” (*Tevenvirinae*) and “probable high transduction frequency”: (*Krishvirus*, *Pseudotevenvirus*, *Kuttervirus*, *Taipeivirus* and *Aglimvirinae*). Therefore, coliphages with probable low transduction frequency, *Tevenvirinae*, may be safer for therapeutic purposes from the viewpoint of their involvement in horizontal gene transfer. The phages of the genera *Krishvirus*, *Pseudotevenvirus*, *Kuttervirus*, *Taipeivirus* and *Aglimvirinae* may be less safe. In our opinion, the conclusions of our bioinformatics analysis are confirmed by the currently available experimental data on the frequencies of plasmid transduction by *Krishvirus*, *Pseudotevenvirus* and *Kuttervirus* coliphages [22,24,62,65]. The approach suggested seems reliable and with enough perspective to be used on various T4-related viruses: not only coliphages, but phages infecting other bacteria as well. It is also possible that the genes we identified as transduction reducing are not the only ones that affect the frequency of gene transfer, and that the transduction frequency is influenced by the recipient strain. The problem of generalized transduction by lytic phages should receive more attention; both bioinformatics and experimental studies are necessary to identify the genetic determinants of transduction and exclude unsafe phages from the list of candidates for the therapy of bacterial infections.

## Figures and Tables

**Table 1:** The genes of T4-related phages that can influence their transduction frequency.

Gene	Functions	Transduction (Integration) Boost in Case of Deletion/Mutation *
*denB*	Endonuclease IV; hydrolysis of single-stranded cytosine-containing DNA	10-fold or more; colonies of transductant bacteria can be obtained ^1^
*alc*	Site-specific termination factor for cytosine-containing (host) DNA, causing premature termination of its transcription [50]	100-fold in *ndd*/*denB*-deficient mutants ^1^
*denA*	Endonuclease II, hydrolysis of cytosine-containing double-stranded DNA	2–5-fold for plasmid integration [40] ^2^
*hmc* and *dCTPases-dUTPase*	HmC-transferase and dCTPase-dUTPase; HmC synthesis	100-fold in *ndd*/*denB*-deficient mutants ^1^
*ndd*	Disrupting structure of *E. coli* chromosome without cleaving or degrading DNA	10-fold or more; colonies of transductant bacteria can be obtained ^1^

^1^—Donor strain: *E. coli* QD JC411; recipient strain: *E. coli* JC411. ^2^—Donor strain: *E. coli* AB1 or MCS1; recipient strain: *E. coli* BE or CR63. * the transduction marker for all experiment was *Arg.*

**Table 2:** The number of homologous clusters versus the percentage of genomes.

Genomes, % of Total	Number of Clusters
0–10	1339
10–25	67
25–50	45
50–75	42
75–90	80
90–95	17
95–100	91

**Table 3:** Occurrence of transduction-reducing genes in the genomes of TerS-containing T4-related coliphages.

Gene	Number of Genomes
*denB*	372
*alc*	372
*denA*	412
*Hmc*	353
*ndd*	376
*dCTPases-dUTPase*	412

**Table 4:** Presence/absence of genes affecting the transduction frequency in individual taxonomic groups of selected viruses.

Family	Subfamily	Genus	Presence (+) or Absence (−) of Genes Influencing Transduction Frequency						Probable Transduction Frequency: High (+), Low (−)
			*dCTPase-dUTPase*	*hmC*	*denB*	*denA*	*ndd*	*alc*	
*Straboviridae*	*Tevenvirinae*	*Dhakavirus*	+	+	+	+	+	+	−
		*Gaprivervirus*	+	−	+	+	+	+	−
		*Kagamiyamavirus*	+	+	+	+	+	+	−
		*Karamvirus*	+	+	+	+	+	+	−
		*Mosigvirus*	+	+	+	+	+	+	−
		*Tequatrovirus*	+	+	+	+	+	+	−
	n/d	*Pseudotevenvirus*	+	−	−	+	+	−	+
	n/d	*Krischvirus*	+	−	−	+	−	−	+
*Ackermannviridae*	*Cvivirinae*	*Kuttervirus*	−	−	−	−	−	−	+
	n/d	*Taipeivirus*	−	−	−	−	−	−	+
	*Aglimvirinae*	n/d	−	−	−	−	−	−	+

## Data Availability

Supporting data are available in the Supplementary Materials.

## Supplementary Materials

The following supporting information can be downloaded at https://www.mdpi.com/article/10.3390/v17050701/s1, Figure S1: The phylogenetic tree of the TerS of selected phages with labeled SH-aLRT support/ultrafast bootstrap support; File S1: Multiple alignment (MUSCLE algorithm) of the TerS of selected phages; Table S1: List of selected phages, includes accession number, taxonomy (genus, subfamily, family), presence of genes influencing transduction frequency, and possible presence of high/low transduction frequency; n/d—not detected; Table S2: Homologs of genes defined as components of the softcore genome of the viruses studied presents genes that occur in 95–100% of genomes (such genes represent the softcore genome), includes other homologs with functions that do not relate to the mechanisms highlighted in the table, as well as homologs with not entirely clear functions, i.e., hypothetical homologs. The number of genomes in which homologs were found is indicated in brackets.

## References

[1] Yang S.-C., Lin C.-H., Aljuffali I.A., Fang J.-Y. (2017). Current Pathogenic Escherichia Coli Foodborne Outbreak Cases and Therapy Development. Arch. Microbiol..

[2] Nasrollahian S., Graham J.P., Halaji M. (2024). A Review of the Mechanisms That Confer Antibiotic Resistance in Pathotypes of *E. coli*. Front. Cell. Infect. Microbiol..

[3] Ahmed S.K., Hussein S., Qurbani K., Ibrahim R.H., Fareeq A., Mahmood K.A., Mohamed M.G. (2024). Antimicrobial Resistance: Impacts, Challenges, and Future Prospects. J. Med. Surg. Public Health.

[4] Pokharel P., Dhakal S., Dozois C.M. (2023). The Diversity of Escherichia Coli Pathotypes and Vaccination Strategies against This Versatile Bacterial Pathogen. Microorganisms.

[5] Wang Y., Subedi D., Li J., Wu J., Ren J., Xue F., Dai J., Barr J.J., Tang F. (2022). Phage Cocktail Targeting STEC O157:H7 Has Comparable Efficacy and Superior Recovery Compared with Enrofloxacin in an Enteric Murine Model. Microbiol. Spectr..

[6] Pirnay J.-P., Blasdel B.G., Bretaudeau L., Buckling A., Chanishvili N., Clark J.R., Corte-Real S., Debarbieux L., Dublanchet A., De Vos D. (2015). Quality and Safety Requirements for Sustainable Phage Therapy Products. Pharm. Res..

[7] Cui Z., Guo X., Feng T., Li L. (2019). Exploring the Whole Standard Operating Procedure for Phage Therapy in Clinical Practice. J. Transl. Med..

[8] Chiang Y.N., Penadés J.R., Chen J. (2019). Genetic Transduction by Phages and Chromosomal Islands: The New and Noncanonical. PLOS Pathog..

[9] Borodovich T., Shkoporov A.N., Ross R.P., Hill C. (2022). Phage-Mediated Horizontal Gene Transfer and Its Implications for the Human Gut Microbiome. Gastroenterol. Rep..

[10] Turner D., Shkoporov A.N., Lood C., Millard A.D., Dutilh B.E., Alfenas-Zerbini P., van Zyl L.J., Aziz R.K., Oksanen H.M., Poranen M.M. (2023). Abolishment of Morphology-Based Taxa and Change to Binomial Species Names: 2022 Taxonomy Update of the ICTV Bacterial Viruses Subcommittee. Arch. Virol..

[11] Denou E., Bruttin A., Barretto C., Ngom-Bru C., Brüssow H., Zuber S. (2009). T4 Phages against *Escherichia coli* Diarrhea: Potential and Problems. Virology.

[12] Sarker S.A., Sultana S., Reuteler G., Moine D., Descombes P., Charton F., Bourdin G., McCallin S., Ngom-Bru C., Neville T. (2016). Oral Phage Therapy of Acute Bacterial Diarrhea With Two Coliphage Preparations: A Randomized Trial in Children From Bangladesh. eBioMedicine.

[13] Nicolas M., Trotereau A., Culot A., Moodley A., Atterbury R., Wagemans J., Lavigne R., Velge P., Schouler C. (2023). Isolation and Characterization of a Novel Phage Collection against Avian-Pathogenic Escherichia Coli. Microbiol. Spectr..

[14] Huff W.E., Huff G.R., Rath N.C., Donoghue A.M. (2013). Method of Administration Affects the Ability of Bacteriophage to Prevent Colibacillosis in 1-Day-Old Broiler Chickens1. Poult. Sci..

[15] Huff W.E., Huff G.R., Rath N.C., Donoghue A.M. (2006). Evaluation of the Influence of Bacteriophage Titer on the Treatment of Colibacillosis in Broiler Chickens1. Poult. Sci..

[16] Sabouri S., Sepehrizadeh Z., Amirpour-Rostami S., Skurnik M. (2017). A Minireview on the *in vitro* and *in vivo* Experiments with Anti-*Escherichia coli* O157:H7 Phages as Potential Biocontrol and Phage Therapy Agents. Int. J. Food Microbiol..

[17] Al-Anany A.M., Hooey P.B., Cook J.D., Burrows L.L., Martyniuk J., Hynes A.P., German G.J. (2023). Phage Therapy in the Management of Urinary Tract Infections: A Comprehensive Systematic Review. Phage.

[18] Bhargava K., Nath G., Dhameja N., Kumar R., Aseri G.K., Jain N. (2023). Bacteriophage Therapy for *Escherichia coli*-Induced Urinary Tract Infection In Rats. Future Microbiol..

[19] Leitner L., Ujmajuridze A., Chanishvili N., Goderdzishvili M., Chkonia I., Rigvava S., Chkhotua A., Changashvili G., McCallin S., Schneider M.P. (2021). Intravesical Bacteriophages for Treating Urinary Tract Infections in Patients Undergoing Transurethral Resection of the Prostate: A Randomised, Placebo-Controlled, Double-Blind Clinical Trial. Lancet Infect. Dis..

[20] Terwilliger A., Clark J., Karris M., Hernandez-Santos H., Green S., Aslam S., Maresso A. (2021). Phage Therapy Related Microbial Succession Associated with Successful Clinical Outcome for a Recurrent Urinary Tract Infection. Viruses.

[21] Tanyashin V.I., Zimin A.A., Boronin A.M. (2003). The Cotransduction of pET System Plasmids by Mutants of T4 and RB43 Bacteriophages. Microbiology.

[22] Tanyashin V.I., Zimin A.A., Shlyapnikov M.G., Boronin A.M. (2003). Transduction of Plasmid Antibiotic Resistance Determinants with PseudoT-Even Bacteriophages. Russ. J. Genet..

[23] Young K.K., Edlin G. (1983). Physical and Genetical Analysis of Bacteriophage T4 Generalized Transduction. Mol. Gen. Genet..

[24] Nikulina A.N., Nikulin N.A., Zimin A.A. (2024). A Study of the Effects of Physico-Chemical Factors on the Frequency of Plasmid Transduction by Bacteriophage RB49. Biophysics.

[25] Rao V.B., Thaker V., Black L.W. (1992). A Phage T4 in Vitro Packaging System for Cloning Long DNA Molecules. Gene.

[26] Black L.W. (2015). Old, New, and Widely True: The Bacteriophage T4 DNA Packaging Mechanism. Virology.

[27] Rao V.B., Fokine A., Fang Q., Shao Q. (2023). Bacteriophage T4 Head: Structure, Assembly, and Genome Packaging. Viruses.

[28] Gao S., Zhang L., Rao V.B. (2016). Exclusion of Small Terminase Mediated DNA Threading Models for Genome Packaging in Bacteriophage T4. Nucleic Acids Res..

[29] Lokareddy R.K., Hou C.-F.D., Li F., Yang R., Cingolani G. (2022). Viral Small Terminase: A Divergent Structural Framework for a Conserved Biological Function. Viruses.

[30] Petrov V.M., Ratnayaka S., Nolan J.M., Miller E.S., Karam J.D. (2010). Genomes of the T4-Related Bacteriophages as Windows on Microbial Genome Evolution. Virol. J..

[31] Kutter E., Bryan D., Ray G., Brewster E., Blasdel B., Guttman B. (2018). From Host to Phage Metabolism: Hot Tales of Phage T4’s Takeover of *E. coli*. Viruses.

[32] Takahashi H., Saito H. (1982). Mechanism of pBR322 Transduction Mediated by Cytosine-Substituting T4 Bacteriophage. Mol. Gen. Genet..

[33] Wilson G.G., Young K.Y., Edlin G.J., Konigsberg W. (1979). High-Frequency Generalised Transduction by Bacteriophage T4. Nature.

[34] Young K.K., Edlin G.J., Wilson G.G. (1982). Genetic Analysis of Bacteriophage T4 Transducing Bacteriophages. J. Virol..

[35] Takahashi H., Saito H. (1982). High-Frequency Transduction of pBR322 by Cytosine-Substituted T4 Bacteriophage: Evidence for Encapsulation and Transfer of Head-to-Tail Plasmid Concatemers. Plasmid.

[36] Bautz F.A., Bautz E.K.F. (1967). Mapping of Deletions in a Non-Essential Region of the Phage T4 Genome. J. Mol. Biol..

[37] Benzer S. (1955). Fine structure of a genetic region in bacteriophage. Proc. Natl. Acad. Sci. USA.

[38] Depew R.E., Snopek T.J., Cozzarelli N.R. (1975). Characterization of a New Class of Deletions of the D Region of the Bacteriophage T4 Genome. Virology.

[39] Morton D., Kutter E.M., Guttman B.S. (1978). Synthesis of T4 DNA and Bacteriophage in the Absence of dCMP Hydroxymethylase. J. Virol..

[40] Selick H.E., Kreuzer K.N., Alberts B.M. (1988). The Bacteriophage T4 Insertion/Substitution Vector System. A Method for Introducing Site-Specific Mutations into the Virus Chromosome. J. Biol. Chem..

[41] Bouras G., Nepal R., Houtak G., Psaltis A.J., Wormald P.-J., Vreugde S. (2023). Pharokka: A Fast Scalable Bacteriophage Annotation Tool. Bioinformatics.

[42] Bayliss S.C., Thorpe H.A., Coyle N.M., Sheppard S.K., Feil E.J. (2019). PIRATE: A Fast and Scalable Pangenomics Toolbox for Clustering Diverged Orthologues in Bacteria. Gigascience.

[43] Okonechnikov K., Golosova O., Fursov M., UGENE team (2012). Unipro UGENE: A Unified Bioinformatics Toolkit. Bioinformatics.

[44] Minh B.Q., Schmidt H.A., Chernomor O., Schrempf D., Woodhams M.D., von Haeseler A., Lanfear R. (2020). IQ-TREE 2: New Models and Efficient Methods for Phylogenetic Inference in the Genomic Era. Mol. Biol. Evol..

[45] Minh B.Q., Nguyen M.A.T., von Haeseler A. (2013). Ultrafast Approximation for Phylogenetic Bootstrap. Mol. Biol. Evol..

[46] Hoang D.T., Chernomor O., von Haeseler A., Minh B.Q., Vinh L.S. (2018). UFBoot2: Improving the Ultrafast Bootstrap Approximation. Mol. Biol. Evol..

[47] Guindon S., Dufayard J.-F., Lefort V., Anisimova M., Hordijk W., Gascuel O. (2010). New Algorithms and Methods to Estimate Maximum-Likelihood Phylogenies: Assessing the Performance of PhyML 3.0. Syst. Biol..

[48] Shannon P., Markiel A., Ozier O., Baliga N.S., Wang J.T., Ramage D., Amin N., Schwikowski B., Ideker T. (2003). Cytoscape: A Software Environment for Integrated Models of Biomolecular Interaction Networks. Genome Res..

[49] Miller E.S., Kutter E., Mosig G., Arisaka F., Kunisawa T., Rüger W. (2003). Bacteriophage T4 Genome. Microbiol. Mol. Biol. Rev..

[50] Drivdahl R.H., Kutter E.M. (1990). Inhibition of Transcription of Cytosine-Containing DNA in vitro by the Alc Gene Product of Bacteriophage T4. J. Bacteriol..

[51] Noguchi T., Takahashi H. (1993). Transactivation of a Plasmid-Borne Bacteriophage T4 Late Gene. Molec. Gen. Genet..

[52] Bouet J.Y., Krisch H.M., Louarn J.M. (1998). Ndd, the Bacteriophage T4 Protein That Disrupts the Escherichia Coli Nucleoid, Has a DNA Binding Activity. J. Bacteriol..

[53] Hirano N., Ohshima H., Sakashita H., Takahashi H. (2007). The Ser176 of T4 Endonuclease IV Is Crucial for the Restricted and Polarized dC-Specific Cleavage of Single-Stranded DNA Implicated in Restriction of dC-Containing DNA in Host Escherichia Coli. Nucleic Acids Res..

[54] Mattson T., Van Houwe G., Bolle A., Epstein R. (1983). Fate of Cloned Bacteriophage T4 DNA after Phage T4 Infection of Clone-Bearing Cells. J. Mol. Biol..

[55] Nikulin N.A., Zimin A.A. (2021). Influence of Non-Canonical DNA Bases on the Genomic Diversity of Tevenvirinae. Front. Microbiol..

[56] Lin H., Simon M.N., Black L.W. (1997). Purification and Characterization of the Small Subunit of Phage T4 Terminase, Gp16, Required for DNA Packaging. J. Biol. Chem..

[57] Lin H., Black L.W. (1998). DNA Requirements in Vivo for Phage T4 Packaging. Virology.

[58] Kreuzer K.N., Yap W.Y., Menkens A.E., Engman H.W. (1988). Recombination-Dependent Replication of Plasmids during Bacteriophage T4 Infection. J. Biol. Chem..

[59] Kreuzer K.N., Saunders M., Weislo L.J., Kreuzer H.W. (1995). Recombination-Dependent DNA Replication Stimulated by Double-Strand Breaks in Bacteriophage T4. J. Bacteriol..

[60] Bryson A.L., Hwang Y., Sherrill-Mix S., Wu G.D., Lewis J.D., Black L., Clark T.A., Bushman F.D. (2015). Covalent Modification of Bacteriophage T4 DNA Inhibits CRISPR-Cas9. mBio.

[61] Matilla M.A., Fang X., Salmond G.P.C. (2014). Viunalikeviruses Are Environmentally Common Agents of Horizontal Gene Transfer in Pathogens and Biocontrol Bacteria. ISME J..

[62] Sørensen A.N., Brøndsted L. (2024). Renewed Insights into Ackermannviridae Phage Biology and Applications. npj Viruses.

[63] Lee Y.-J., Dai N., Walsh S.E., Müller S., Fraser M.E., Kauffman K.M., Guan C., Corrêa I.R., Weigele P.R. (2018). Identification and Biosynthesis of Thymidine Hypermodifications in the Genomic DNA of Widespread Bacterial Viruses. Proc. Natl. Acad. Sci. USA.

[64] Majewska J., Beta W., Lecion D., Hodyra-Stefaniak K., Kłopot A., Kaźmierczak Z., Miernikiewicz P., Piotrowicz A., Ciekot J., Owczarek B. (2015). Oral Application of T4 Phage Induces Weak Antibody Production in the Gut and in the Blood. Viruses.

[65] Matilla M.A., Salmond G.P.C. (2014). Bacteriophage ϕMAM1, a Viunalikevirus, Is a Broad-Host-Range, High-Efficiency Generalized Transducer That Infects Environmental and Clinical Isolates of the Enterobacterial Genera Serratia and Kluyvera. Appl. Environ. Microbiol..

[66] Calero-Cáceres W., Ye M., Balcázar J.L. (2019). Bacteriophages as Environmental Reservoirs of Antibiotic Resistance. Trends Microbiol..

[67] Doub J.B. (2021). Risk of Bacteriophage Therapeutics to Transfer Genetic Material and Contain Contaminants Beyond Endotoxins with Clinically Relevant Mitigation Strategies. Infect. Drug Resist..

[68] Kosmopoulos J.C., Campbell D.E., Whitaker R.J., Wilbanks E.G. (2023). Horizontal Gene Transfer and CRISPR Targeting Drive Phage-Bacterial Host Interactions and Coevolution in “Pink Berry” Marine Microbial Aggregates. Appl. Environ. Microbiol..

[69] Keen E.C., Bliskovsky V.V., Malagon F., Baker J.D., Prince J.S., Klaus J.S., Adhya S.L. (2017). Novel “Superspreader” Bacteriophages Promote Horizontal Gene Transfer by Transformation. mBio.

[70] Kreuzer K.N., Brister J.R. (2010). Initiation of Bacteriophage T4 DNA Replication and Replication Fork Dynamics: A Review in the Virology Journal Series on Bacteriophage T4 and Its Relatives. Virol. J..

[71] Dedrick R.M., Guerrero-Bustamante C.A., Garlena R.A., Russell D.A., Ford K., Harris K., Gilmour K.C., Soothill J., Jacobs-Sera D., Schooley R.T. (2019). Engineered Bacteriophages for Treatment of a Patient with a Disseminated Drug-Resistant Mycobacterium Abscessus. Nat. Med..

