# Supplementary material for: Treatment of E. coli Infections with T4-Related Bacteriophages Belonging to Class Caudoviricetes: Selecting Phage on the Basis of Their Generalized Transduction Capability
Source: Viruses. 2025 May 14;17(5):701. doi: 10.3390/v17050701 (PMC12115623; doi:10.3390/v17050701)
Supplement: Supplementary file 1 [file viruses-17-00701-s001.zip › Table S2 Homologs of genes defined as components of the softcore genome of the viruses .pdf]

Table S2. Homologs of genes identified as components of the softcore genome of the viruses studied.

Other – homologs with functions that do not relate to the mechanisms highlighted in the table, as well as homologs with unclear functions, hypothetical homologs. The number of genomes in which homologs were found is given in brackets.

DNA replication, repair, recombination and gene expression
subunit of clamp loader of DNA polymerase (431), RegA translation repressor of early genes (431), sliding clamp DNA polymerase accessory protein (431), DNA helicase (430), DNA primase (430), DnaB-like replicative helicase (430), SbcC-like subunit of palindrome specific endonuclease (430), single strand DNA binding protein (430), UvsX-like recombinase (430), subunit of clamp loader of DNA polymerase (429), DNA polymerase (429), DNA topoisomerase II medium subunit (429), ATP-dependent DNA ligase (428), FmdB-like transcriptional regulator (418), anaerobic glutaredoxin nrdH (412), ATP-dependent DNA helicase uvsW (412), Dda-like helicase (412), RNA ligase A (412), RNA polymerase binding protein RpbA (412), RNA polymerase sigma factor (412), Srd anti-sigma factor (412), UvsY-like recombination mediator (412), DNA helicase loader (411), late promoter transcriptional regulator (411), ribonuclease H (411), DNA topoisomerase II large subunit (410).
Auxiliary metabolism
ribonucleoside-diphosphate reductase alpha subunit NrdA (430), ribonucleoside-diphosphate reductase small subunit NrdB (430), dCMP deaminase (425), dCTPase/dUTPase (412), endonuclease II (DenA) (412), exonuclease DexA (412), thioredoxin NrdC (412), deoxynucleoside monophosphate kinase (411), polynucleotide kinase (411), dihydrofolate reductase (410), nucleotidyltransferase (410), thymidine kinase (410).
Morphogenesis
baseplate hub subunit and lysozyme (431), baseplate wedge subunit (431), baseplate wedge subunit (431), head closure (431), head closure Hc2 (431), head scaffolding protein (431), head-tail adaptor Ad2 (431), portal protein (431), tail tube protein (431), major head protein (431), terminase large subunit (431), terminase small subunit (431), tail completion and sheath stabilizer protein (430), tail sheath stabilizer (430), baseplate wedge subunit (429), head maturation protease (429), tail sheath (429), baseplate hub assembly protein (412), baseplate hub distal subunit (412), head scaffolding protein (412), head vertex assembly chaperone (412), head vertex protein (412), prohead (412), tail fiber chaperone (412), tail tube (412), long tail fiber protein proximal connector (412), minor head protein inhibitor of protease (412), long tail fiber protein distal subunit (412), baseplate hub (411), baseplate hub subunit (411), baseplate tail tube cap (411), baseplate wedge subunit (411), baseplate wedge subunit (411), baseplate wedge subunit (411), baseplate wedge tail fiber protein connector (411), fibritin neck

whisker (411), head assembly chaperone protein (411), holin (411), tail collar fiber protein (411), baseplate wedge subunit (410).
Other
phosphatase (427), Anti-CBASS protein Acb1 (422), RIIA lysis inhibitor (421), Rz-like spanin (411), Rz-like spanin (411), rI lysis inhibition regulator (410), hypothetical protein (429), hypothetical protein (417), hypothetical protein (412), hypothetical protein (412), hypothetical protein (412), hypothetical protein (412), hypothetical protein (411).
