# Supplementary material for: Treatment of E. coli Infections with T4-Related Bacteriophages Belonging to Class Caudoviricetes: Selecting Phage on the Basis of Their Generalized Transduction Capability
Source: Viruses. 2025 May 14;17(5):701. doi: 10.3390/v17050701 (PMC12115623; doi:10.3390/v17050701)

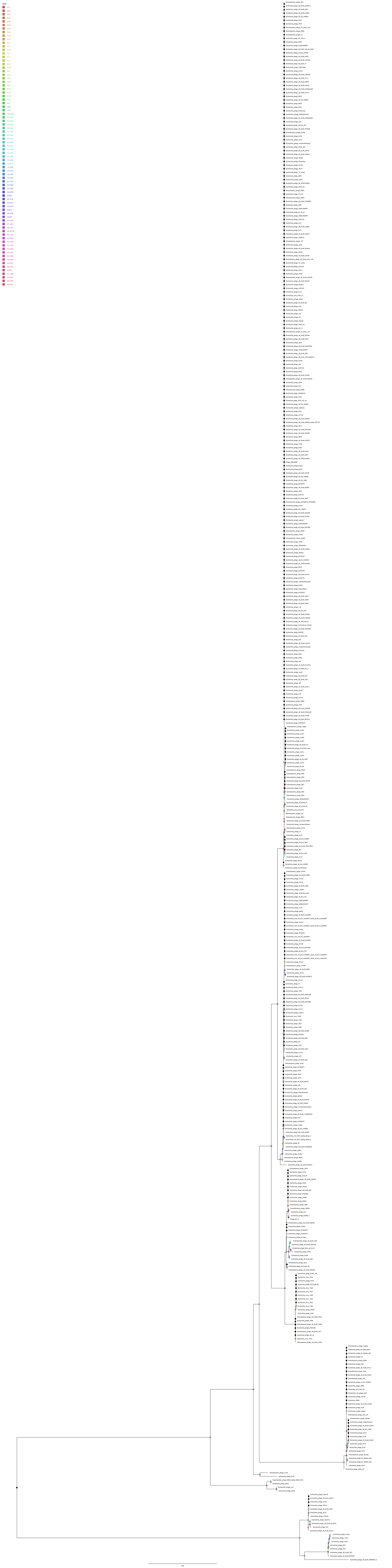

Supplement: Supplementary file 1 [file figure S1 Phylogenetic tree of TerS.pdf › figure S1 Phylogenetic tree of TerS.pdf]
